# National mortality burden due to communicable, non-communicable, and other diseases in Ethiopia, 1990–2015: findings from the Global Burden of Disease Study 2015

**DOI:** 10.1186/s12963-017-0145-1

**Published:** 2017-07-21

**Authors:** Awoke Misganaw, Tilahun N. Haregu, Kebede Deribe, Gizachew Assefa Tessema, Amare Deribew, Yohannes Adama Melaku, Azmeraw T. Amare, Semaw Ferede Abera, Molla Gedefaw, Muluken Dessalegn, Yihunie Lakew, Tolesa Bekele, Mesoud Mohammed, Biruck Desalegn Yirsaw, Solomon Abrha Damtew, Kristopher J. Krohn, Tom Achoki, Jed Blore, Yibeltal Assefa, Mohsen Naghavi

**Affiliations:** 1 Institute for Health Metrics and Evaluation, University of Washington, Seattle, USA; 2 Africa Population and Health Research Center, Nairobi, Kenya; 3 Brighton and Sussex Medical School, Brighton, UK; 4 School of Public Health, Addis Ababa University, Addis Ababa, Ethiopia; 5 School of Public Health, University of Adelaide, Adelaide, Australia; 6 Department of Reproductive Health, Institute of Public Health, University of Gondar, Gondar, Ethiopia; 7 KEMRI-Wellcome Trust Research Programme, Kilifi, Kenya; 8 Nuffield Department of Clinical Medicine, University of Oxford, Oxford, UK; 9 St. Paul Millennium Medical College, Addis Ababa, Ethiopia; 10 School of Public Health, College of Health Sciences, Mekelle University, Mekelle, Ethiopia; 11 Federal Ministry of Health, Addis Ababa, Ethiopia; 12 College of Medicine and Health Sciences, Bahir Dar University, Bahir Dar, Ethiopia; 13 Institute for Biological Chemistry and Nutrition, University of Hohenheim, Stuttgart, Germany; 14 Amref Health Africa in Ethiopia, Addis Ababa, Ethiopia; 15 Ethiopian Public Health Association, Addis Ababa, Ethiopia; 16 Department of Public Health, College of Medicine and Health Sciences, Madda Walabu University, Bale Robe, Ethiopia; 17 University of South Australia, Adelaide, Australia; 18 College of Health Sciences and Medicine, Wolayta Sodo University, Sodo, Ethiopia; 19 University of Queensland, Brisbane, Australia

**Keywords:** Causes of death, Mortality, Communicable disease, Non-communicable diseases, Ethiopia, Global burden of disease

## Abstract

**Background:**

Ethiopia lacks a complete vital registration system that would assist in measuring disease burden and risk factors. We used the Global Burden of Diseases, Injuries, and Risk Factors Study 2015 (GBD 2015) estimates to describe the mortality burden from communicable, non-communicable, and other diseases in Ethiopia over the last 25 years.

**Methods:**

GBD 2015 mainly used cause of death ensemble modeling to measure causes of death by age, sex, and year for 195 countries. We report numbers of deaths and rates of years of life lost (YLL) for communicable, maternal, neonatal, and nutritional (CMNN) disorders, non-communicable diseases (NCDs), and injuries with 95% uncertainty intervals (UI) for Ethiopia from 1990 to 2015.

**Results:**

CMNN causes of death have declined by 65% in the last two-and-a-half decades. Injury-related causes of death have also decreased by 70%. Deaths due to NCDs declined by 37% during the same period. Ethiopia showed a faster decline in the burden of four out of the five leading causes of age-standardized premature mortality rates when compared to the overall sub-Saharan African region and the Eastern sub-Saharan African region: lower respiratory infections, tuberculosis, HIV/AIDS, and diarrheal diseases; however, the same could not be said for ischemic heart disease and other NCDs. Non-communicable diseases, together, were the leading causes of age-standardized mortality rates, whereas CMNN diseases were leading causes of premature mortality in 2015. Although lower respiratory infections, tuberculosis, and diarrheal disease were the leading causes of age-standardized death rates, they showed major declines from 1990 to 2015. Neonatal encephalopathy, iron-deficiency anemia, protein-energy malnutrition, and preterm birth complications also showed more than a 50% reduction in burden. HIV/AIDS-related deaths have also decreased by 70% since 2005. Ischemic heart disease, hemorrhagic stroke, and ischemic stroke were among the top causes of premature mortality and age-standardized death rates in Ethiopia in 2015.

**Conclusions:**

Ethiopia has been successful in reducing deaths related to communicable, maternal, neonatal, and nutritional deficiency diseases and injuries by 65%, despite unacceptably high maternal and neonatal mortality rates. However, the country’s performance regarding non-communicable diseases, including cardiovascular disease, diabetes, cancer, and chronic respiratory disease, was minimal, causing these diseases to join the leading causes of premature mortality and death rates in 2015. While the country is progressing toward universal health coverage, prevention and control strategies in Ethiopia should consider the double burden of common infectious diseases and non-communicable diseases: lower respiratory infections, diarrhea, tuberculosis, HIV/AIDS, cardiovascular disease, cancer, and diabetes. Prevention and control strategies should also pay special attention to the leading causes of premature mortality and death rates caused by non-communicable diseases: cardiovascular disease, cancer, and diabetes. Measuring further progress requires a data revolution in generating, managing, analyzing, and using data for decision-making and the creation of a full vital registration system in the country.

## Background

The 2007 Population and Housing Census projection estimated that Ethiopia would have a total population of 90 million in 2015 [1]. Nearly half (45%) of the population is under the age of 15 years, only 3% are above the age of 65, and the sex ratio is almost equal [1]. Health policy in Ethiopia focuses on the promotion, prevention, and control of diseases and injuries. In 2015 the country finalized a 20-year National Health Sector Development Program (HSDP) [2] and started the Health Sector Transformation Plan (HSTP). The HSDP was launched in 1997 and has been implemented in four phases focusing on prioritized disease prevention, decentralization of the delivery of health services, strengthening partnerships between the government and non-governmental organizations to implement basic health care packages and achieve universal primary health care coverage, and increased national health spending [2]. And, more recently, HSTP is aiming to improve quality and equity of health services and universal health coverage between 2016 and 2020 [3]**.**

Recent studies indicate that Ethiopia has achieved significant results in improving child survival, reducing maternal mortality and total fertility rates, and increasing life expectancy [4–6]. The estimated average life expectancy at birth has increased from 45.5 years in 1990 to 61.7 years in 2013, under-5 mortality has decreased by two-thirds, the maternal mortality ratio decreased from 708 per 100,000 live births in 1990 to 497.4 in 2013, and the total fertility rate decreased from 7.2 in 1990 to 4.5 in 2013 [7–10]. Overall deaths due to HIV, tuberculosis, and malaria have also decreased in Ethiopia [5, 11].

Communicable diseases, nutritional deficiencies, and maternal and neonatal mortality have been competing priorities with the limited resources in the HSDP era; however, non-communicable diseases were less-prioritized Ethiopian public health problems until recently [12, 13]. The national envisioning plan has targeted an age-standardized mortality rate of 509 per 100,000 population due to non-communicable diseases and decreased rates for HIV, tuberculosis, and malaria by 2035: 6, 3, and 0, respectively [12].

Lack of reliable data sources has been a longstanding obstacle to guiding health policies to measure the performance of the Health Sector Development Program (HSDP) and create benchmarks for newly developed policy frameworks, strategies, and targets in Ethiopia [12]. In order to create policy frameworks and envision targets, the country used different death registration scenarios based upon lower-middle- and upper-middle-income countries since the vital events registration system has just begun to function in Ethiopia [12]. Thus far, limited verbal autopsy (VA) studies from demographic surveillance sites and urban-based mortality surveillance programs, health care facility reports, and surveys have been used to measure HSDP’s progress and to demonstrate the emerging challenges of non-communicable diseases in the country [14, 15].

In this context, the results of the Global Burden of Diseases, Injuries, and Risk Factors Study 2015 (GBD 2015) provide an additional opportunity to examine trends in the country’s health profile between 1990 and 2015. The results can assist in tracking the progress of the Health Sector Transformation Plan (HSTP) from 2016 to 2020 in Ethiopia, which is in line with the Sustainable Development Goals [12]. GBD data contain all the required information to compare health profiles at the country level. Hence, we used GBD 2015 results for Ethiopia to examine the performance of its health system in terms of mortality due to diseases and injuries between 1990 and 2015.

## Methods

### Data sources

GBD 2015 data were used to analyze the number of deaths and years of life lost (YLL) for Ethiopia. GBD 2015 utilizes comprehensive sources of data that meet inclusionary criteria and rigorous analysis to estimate trends of cause-specific mortality rates for 195 countries [16]. It identifies all available data sources, evaluates the quality, and corrects for known bias in each data source [7]. Data were adjusted for garbage coding and misclassification of causes of deaths. Garbage coding is the assignment of causes of death that are not the underlying cause of death [17]. The effect of garbage coding in the estimation of causes of death is usually high for countries that use vital registration and very low for countries using verbal autopsy. GBD 2015 used 171 site-years of cause of death data sources; these sources were used to produce national estimates for Ethiopia. Of the 171 site-year sources, 70 site-year were nationally representative and 52 site-year represented subnational locations in Ethiopia. Approximately 102 site-year data sources were from sibling history, an indirect method exploring maternal mortality through surviving siblings, and 63 site-years of data sources used verbal autopsy data collection techniques, the method of ascertaining cause of death from information obtained from relatives or associates of a deceased person [18]. Data were relatively scarce for Ethiopia; when this occurs, GBD uses two additional approaches to estimate causes of death. The first approach involves identifying covariates for each cause which are used to provide better estimates; the second borrows strength from the regional and super-regional model to produce estimates. The Supplementary Appendix provides further details on data sources [16].

### Causes of death modeling

The GBD approach to estimate all-cause mortality and cause-specific mortality rates has been previously described [16, 17]. Causes of death by age, sex, and year were measured mainly using cause of death ensemble modeling (CODEm) [7]. A detailed description of CODEm is reported elsewhere [17]. In brief, CODEm tests a wide range of models, such as mixed effects linear models and spatiotemporal Gaussian process regression (ST-GPR) models, and constructs an ensemble model based on the performance of the different models [17]. Out-of-sample predictive validity testing was used to select the ensemble model for estimation of morality rate [17]. In this model, uncertainty intervals are generated by sampling the posterior distribution of each component model in proportion to the weight of each model in the ensemble. Vital registration and VA data were corrected for garbage codes based on the GBD 2015 algorithm [17].

### Descriptions of scenarios used to estimate rates

GBD 2015 used decomposition analysis to estimate mortality rates and causes of death for each age-sex-year group [9]. Trends were decomposed by the contribution of total increase in population size, aging of the population, and changes in age-specific and sex-specific rates [19, 20]. Two counterfactual sets of cause of death numbers were computed: a population growth scenario was computed as the number of deaths expected in 2015 if only total population numbers increased to the level of 2015 but the age-sex structure of population remained the same as in 1990 and age-sex specific rates remained at 1990 levels; and, two, a population growth and population aging scenario was computed as the number of deaths expected in 2015, using 1990 age-sex specific rates and 2015 age-specific and sex-specific population numbers [20]. Accordingly, three different scenarios are presented as a percentage change compared to 1990. The difference between 1990 numbers and the population growth scenario is the change in death numbers strictly due to growth in the total population. The change from the population growth scenario to the population growth and aging scenario is the number of deaths due to aging of the population. The difference between 2015 deaths, population growth, and aging scenario is the difference in deaths due to epidemiological change in age-specific and sex-specific death rates [7, 20]. While estimating crude death rates, the effect of national population growth during the study period was controlled, and changes in population size and composition were controlled while estimating age-standardized rates [21].

### Interpretation of the results

We followed GBD categorization of diseases and injuries to present and interpret the estimates. GBD uses a hierarchy of mutually exclusive and collectively exhaustive causes that organizes 249 fatal diseases and injuries into four levels [16]. The first level shows three broad categories: communicable, maternal, neonatal, and nutritional (CMNN) disorders, non-communicable diseases (NCDs), and injuries. Level 2 has 21 cause groups, such as cardiovascular diseases, whereas Levels 3 and 4 provide more disaggregated causes [21].We present the estimates in terms of crude death rates, crude YLL rates, age-standardized death rates, and age-standardized YLL rates, with 95% uncertainty intervals (UI). Years of life lost for each cause were estimated by using standard GBD 2015 methodology [7, 17]. This method involves multiplying deaths occurring as a result of a given cause by the reference standard life expectancy at the age when the death occurred, based upon the lowest observed death rate for each 5-year age group for populations greater than 5 million [22]. GBD 2015 produced uncertainty intervals at key steps of all-cause and cause-specific mortality estimation to account for uncertainties that arise from adjusting sources, sample size, and other model specifications [17]. We report positive percentage changes to show increasing trends and negative percentage changes to show decreasing trends of rates from 1990 to 2015.

## Results

### Crude death rates and percentage changes

Overall, 677,045.6 (95% UI: 502,236.1–908,743.0) deaths occurred in Ethiopia in 2015 and 316,969.7 (95% UI: 205,647.4–496,323.5) of these were among females. Crude death rates for broad groups of causes and the crude all-cause mortality rate are presented in Table 1. The crude all-cause mortality rate was 680.9 per 100,000 people (95% UI: 505.1–913.9) in 2015, of which 337 (95% UI: 273.8–421.3) per 100,000 were caused by CMNN diseases and 286.9 (95% UI: 188.1–423.0) per 100,000 were due to NCDs. Overall, the death rate has decreased for the three broad groups of causes from 1990 to 2015; however, CMNN diseases and injuries showed greater reduction of 75% each, while NCDs showed a slower decline of 40% (Table 1).

**Table 1:** Crude death rates (CDR) per 100,000 for both sexes and all age groups from 1990 to 2015

Cause of death	1990		2015		CDR % change
	Number	CDR	Number	CDR	
All cause	952,828.3 (886,385.2–1,024,947)	1989.5 (1850.8–2140.1)	677,045.6 (502,236.1–908,743)	680.9 (505.1–913.9)	−66%
CMNN diseases	614,362.2 (574,223.5–660,499.6)	1282.8 (1199.0–1379.1)	335,020.1 (272,264.7–418,941.7)	337.0 (273.8–421.3)	−74%
HIV/AIDS and tuberculosis	72,587.7 (53,314.7–94,111.0)	151.7 (111.3–196.5)	72,406.8 (50,591.6–100,423.7)	72.8 (50.9–101)	−52%
Diarrhea, lower respiratory infections, and other common infectious diseases	330,664.8 (274,939.7–417,449.7)	690.4 (574.1–871.6)	146,563.8 (113,249.0–189,932.6)	147.4 (113.9–191.0)	−79%
Neglected tropical diseases and malaria	36,616.9 (16,225.2–67,521.0)	76.5 (33.9–141.0)	5154.8 (3128.3–8152.7)	5.18 (3.2–8.2)	−93.2%
Maternal disorders	18,282.8 (13,773–24,091.4)	38.2 (28.8–50.3)	13,016.7 (5496.7–28,518.9)	13.1 (5.5–28.7)	−66%
Neonatal disorders	92,853.9 (79,341.0–105,634.4)	193.9 (165.7–220.6)	61,617.7 (53,179.5–70,111.2)	62.0 (53.5–70.5)	−68%
Nutritional deficiencies	43,604.3 (19,376.4–76,178.0)	91.1 (40.5–159.1)	18,889.2 (11,141.3–29,367.4)	19 (11.2–29.5)	−79%
Other communicable, maternal, neonatal, and nutritional diseases	19,752.0 (10,545.4–32,122.7)	41.2 (22.0–67.1)	17,371.1 (10,558.4–26,368.6)	17.5 (10.6–26.5)	−58%
Non-communicable diseases	227,980.4(196,470.3–260,253.7)	476.0 (410.2–543.4)	285,301.3 (187,026.9–420,603)	286.9 (188.1–423.0)	−39.7%
Neoplasms	37,661.7 (31,487.9–44,630.8)	78.6 (65.7–93.2)	55,306.2 (32,618.6–89,317.6)	55.6 (32.8–89.8)	−29%
Cardiovascular diseases	94,315.8 (80,142.5–109,363.7)	196.9 (167.3–228.4)	119,829.7 (76,656.5–176,502.7)	120.5 (77.1–177.5)	−39%
Chronic respiratory diseases	15,081.4 (12,636.8–17,964.9)	31.5 (26.4–37.5)	14,950.9 (9498.5–22,227.8)	15.0 (9.6–22.4)	−52%
Cirrhosis	14,301.6 (11,304.1–17,231.9)	29.9 (23.6–36.0)	14,112.5 (8297.3–22,590.6)	14.2 (8.3–22.7)	−53%
Digestive diseases	17,830.2 (14,475.0–22,665.0)	37.2 (30.2–47.3)	16,962.0 (10,705.6–25,591.1)	17.1 (10.8–25.7)	−54%
Neurological disorders	6648.9 (5012.6–8589.0)	13.9 (10.5–17.9)	10,663.5 (7062.8–15,629.9)	10.7 (7.1–15.7)	−23%
Mental and substance use disorders	1228.4 (895.5–1697.1)	2.56 (1.9–3.5)	1384.9 (722.1–2587.0)	1.4 (0.7–2.6)	−46%
Diabetes, urogenital, blood, and endocrine diseases	21,161.7 (17,017.8–25,995.9)	44.2 (35.5–54.3)	27,709.6 (17,596.1–42,125.1)	27.9 (17.7–42.4)	−37%
Musculoskeletal disorders	230.7 (173.9–291.0)	0.5 (0.4–0.6)	413.1 (204.8–780.2)	0.42 (0.2–0.8)	−14%
Other non-communicable diseases	19,520.0 (11,678.1–29,509.1)	40.8 (24.4–61.6)	23,968.7(14,157.4–31,544.2)	24.1 (14.2–31.7)	−41%
Injuries	110,485.7 (73,722.94–149,884.4)	230.7 (153.9–313.0)	56,724.24 (37,272.53–85,873.47)	57.1 (37.5–86.4)	−75%
Transport injuries	14,059.3 (9903.0–18,617.9)	29.4 (20.7–38.9)	14,094.9 (8922.1–22,241.4)	14.2 (9.0–22.4)	−52%
Unintentional injuries	35,703.4 (23,064.2–48,263.9)	74.6 (48.2–100.8)	28,785.6 (19,680.0–41,660.1)	29.0 (19.8–41.9)	−61%
Self-harm and interpersonal violence	10,402.9 (7466.3–17,086.6)	21.7 (15.6–35.7)	13,484.4 (7542.0–24,122.4)	13.6 (7.6–24.3)	−38%
Forces of nature, war, and legal intervention	50,320.2 (20,052.2–87,479.3)	105.1 (41.9–182.7)	359.3 (106.6–664.4)	0.4 (0.1–0.7)	100%

In 2015, HIV/AIDS and tuberculosis collectively caused 72.8 (95% UI: 50.9–101.0) deaths per 100,000 people (Table 1). Diarrhea, lower respiratory infections, and other common infectious diseases caused 147.4 (95% UI: 113.9–191.0) deaths per 100,000 people. Cardiovascular diseases and neoplasms caused 120.5 (95% UI: 77.1–177.5) and 55.6 (95% UI: 32.8–89.8) deaths per 100,000 people, respectively (Table 1). Transport injuries and unintentional injuries caused 14.2 (95% UI: 9.0–22.4) and 29.0 (95% UI: 19.8–41.9) deaths per 100,000 people, respectively.

All Level 2 cause categories declined from 1990 to 2015, with a major reduction in CMNN-related causes of death (Table 1). The top two leading causes of death that showed decline were HIV/AIDS with tuberculosis, which decreased by 52%, and diarrhea, lower respiratory infections, and other common infectious diseases, which collectively declined by 79%. The two leading causes among NCDs, cardiovascular diseases and neoplasms, declined by 39 and 29%, respectively. Furthermore, mortality due to unintentional and transport injuries fell 61 and 52%, respectively, between 1990 and 2015.

### Age-standardized death rates and percentage changes

**Fig. 1:**
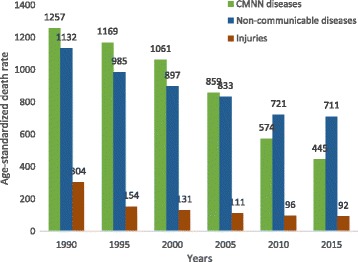
Levels and trends of age-standardized death rates per 100,000 by major causes for both sexes and all age groups in Ethiopia, 1990–2015

The burden from CMNN diseases, NCDs, and injuries, in terms of age-standardized death rates for both sexes and all age groups, is shown in Table 2. Overall, total mortality and age-standardized death rates for each of the broad groups of causes decreased between 1990 and 2015. In 2015, the all-cause age-standardized death rate was 1248.0 per 100,000 people (95% UI: 860.3–1760.3), of which NCDs caused 710.9 (95% UI: 468.8–1036.2) and CMNN diseases caused 445.0 (95% UI: 326.7–600.7) deaths per 100,000 people (Table 2). NCDs showed a smaller reduction of 37% between 1990 and 2015 compared to the 65% decrease for CMNN causes and 70% for injuries (Table 2). Non-communicable diseases were the leading causes of age-standardized death rates, causing 711 deaths per 100,000 people (95% UI: 468.8–1036.2) in 2015, as indicated in Fig. 1.

**Table 2:** Age-standardized death rates (ASDR) per 100,000 for both sexes and all age groups with Level 1 and 2 categories, 1990 to 2015

Causes of death	1990	2015	ASDR % change
All cause	2693.0 (2419.3–2985.2)	1248.0 (860.3–1760.3)	−54%
CMNN diseases	1257.1 (1128.3–1395)	445.0 (326.7–600.7)	−65%
HIV/AIDS and tuberculosis	267.3 (194.1–338.2)	122.0 (79.9–174.8)	−54%
Diarrhea, lower respiratory infection, and other common infectious diseases	674.0 (581.5–789.0)	223.6 (158.0–308.8)	−67%
Neglected tropical diseases and malaria	71.7 (38.7–123.0)	6.0 (3.5–9.8)	−92
Maternal disorders	47.4 (35.7–62.6)	15.0 (6.3–33.9)	−68%
Neonatal disorders	77.5 (66.3–88.3)	36.5 (31.5–41.5)	−53%
Nutritional deficiencies	91.1 (62.3–129.2)	27.8 (17.7–40.5)	−69%
Other communicable, maternal, neonatal, and nutritional diseases	28 (18.5–40.2)	14.1 (9.2–20.1)	−49.6
Non-communicable diseases	1131.6 (981.7–1278.6)	710.9 (468.8–1036.2)	−37%
Neoplasms	176.2 (149.8–204.8)	122.5 (74.3–192.4)	−31%
Cardiovascular diseases	554.3 (476.0–631.4)	350.0 (232.7–500.7)	−37%
Chronic respiratory diseases	81.2 (67.1–95.4)	40.7 (26.4–59.5)	−50%
Cirrhosis	64.7 (52.2–76.8)	31.8 (19.2–50.1)	−51%
Digestive diseases	86.8 (73.5–101.4)	42.8 (27.9–62.7)	−51%
Neurological disorders	38.1 (31.2–45.8)	30.9 (20.5–43.8)	−19%
Mental and substance use disorders	4.4 (3.2–6.0)	2.4 (1.3–4.4)	−47%
Diabetes, urogenital, blood, and endocrine diseases	97.6 (77.9–125.6)	67.4 (42.8–102.4)	−31%
Musculoskeletal disorders	1.0 (0.72–1.4)	0.9 (0.4–1.7)	−15%
Other non-communicable diseases	27.2 (19.3–37.8)	21.5 (13.8–29.1)	−21%
Injuries	304.3 (222.0–390.1)	92.2 (59.1–140.6)	−70%
Transport injuries	41.6 (31.2–53.30)	21.4 (13.4–34.5)	−48%
Unintentional injuries	105.7 (85.8–130.0)	50.1 (32.6–73.4)	−53%
Self-harm and interpersonal violence	33.3 (24.2–50.5)	20.2 (11.6–35.2)	−39%
Forces of nature, war, and legal intervention	123.9 (51.4–205.70)	0.4 (0.1–0.7)	99.7%

Diarrhea, lower respiratory infections, and other common infectious diseases caused 223.6 (95% UI: 158.0–308.8) age-standardized deaths per 100,000 people in 2015, combined. The age-standardized death rate for HIV/AIDS and tuberculosis, combined, was 122.0 (95% UI: 79.9–174.8) deaths per 100,000 people (Table 2). Cardiovascular diseases and neoplasms caused 350.0 (95% UI: 232.7–500.7) and 122.5 (95% UI: 74.3–192.4) deaths per 100,000 people, respectively (Table 2). Transport injuries and unintentional injuries caused 21.4 (95% UI: 13.4–34.5) and 50.1 (95% UI: 32.6–73.4) deaths per 100,000 people, respectively.

From 1990 to 2015, all Level 2 cause categories declined, with a major reduction of CMNN causes of death (Table 2). The combined age-standardized death rate from HIV/AIDS and tuberculosis declined by 54%. Similarly, the death rate from diarrhea, lower respiratory infections, and other common infectious diseases collectively declined 67% (Table 2). The age-standardized death rates from cardiovascular diseases and neoplasms showed 37 and 31% reductions, respectively, between 1990 and 2015. Age-standardized death rates due to injuries also declined, as the rate for unintentional injuries declined by 53% and that for transport injuries by 48% (Table 2).

**Fig. 2:**
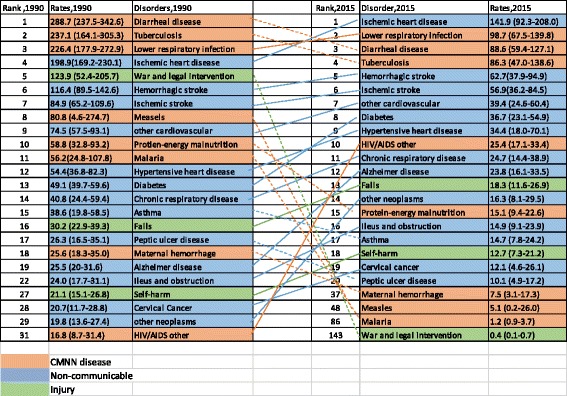
Ranks of age-standardized death rates per 100,000 people for both sexes and all age groups in Ethiopia, 1990–2015

As shown in Fig. 2, the top 10 leading causes accounted for more than 50% of the total age-standardized death rate in 2015. Six of the 10 leading causes were NCDs; however, only two NCDs were in the top five leading causes. The top five leading causes in 2015 were ischemic heart disease, lower respiratory infections, diarrheal diseases, tuberculosis, and hemorrhagic stroke and caused 141.9 (95% UI: 92.3–208.0), 98.7 (95% UI: 67.5–139.8), 88.6 (95% UI: 59.4–127.1), 86.3 (95% UI: 47.0–138.6), and 62.7 (95% UI: 37.9–94.9) deaths per 100,000 people, respectively (Fig. 2 and Table 2).

**Fig. 3:**
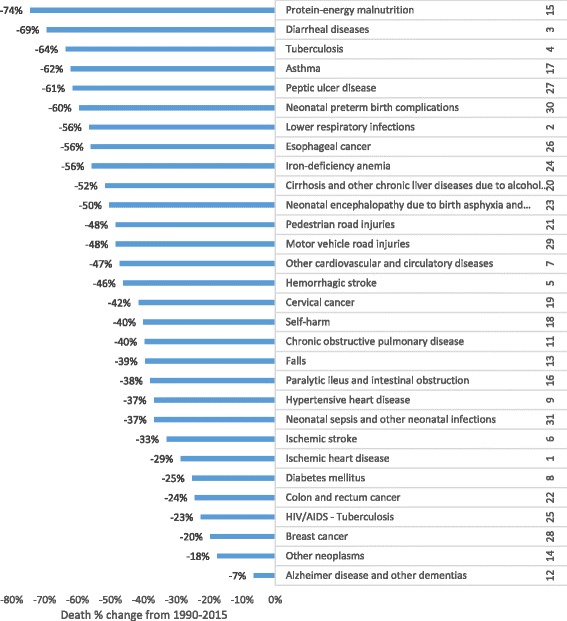
Percentage change in age-standardized death rates for the top 30 causes of death, both sexes and all age groups, between 1990 and 2015;* we excluded HIV/AIDS, the 10th leading cause, and other causes in each disease group

**Fig. 4:**
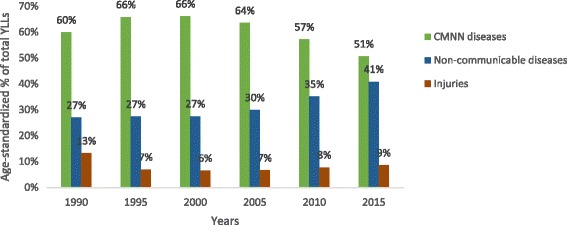
Percentage contribution of total age-standardized YLL rates per 100,000 by years for major causes for both sexes and all age groups in Ethiopia, 1990–2015

The percent change in the age-standardized death rate per 100,000 for the top 30 leading causes of death between 1990 and 2015 is shown in Fig. 3. Significantly large reductions occurred in three of the top five leading causes of death: diarrheal disease (69%), tuberculosis (64%), and lower respiratory infections (56%) (Fig. 4). The first, fifth, and sixth leading causes of death also declined: ischemic heart disease (29%), hemorrhagic stroke (46%), and ischemic stroke (33%) (Fig. 4). Between 1990 and 2005, HIV/AIDS increased by 1013%; however, since 2005 this rate has decreased by 70% (data not shown).

Other causes that declined by 50% or greater are shown in Fig. 3 and include neonatal encephalopathy, iron-deficiency anemia, protein-energy malnutrition, and preterm birth complications.

### Crude YLL rates and percentage changes

In 2015, the all-cause crude YLL rate was 32,397.0 per 100,000 people (95% UI: 26,283.3–40,426.8), of which CMNN diseases caused 20,744.6 (95% UI: 17,785.6–24,205.2) and NCDs caused 8823.0 (95% UI: 5926.8–12,768.2) per 100,000 people (Table 3). The crude YLLs per 100,000 people showed higher reductions for CMNN diseases and injuries – 77 and 79%, respectively – while NCDs declined 47% (Table 3).

**Table 3:** Crude YLL rates per 100,000 peoples for both sexes and all age groups with Level 1 and 2 categories, from 1990 to 2015

Causes of YLLs	1990	2015	Crude YLL % change
All causes	120,333.8 (114,133.0–126,957.4)	32,397.0 (26,283.3–40,426.8)	−73%
CMNN diseases	90,197.3 (84,907.0–96,574.9)	20,744.6 (17,785.6–24,205.2)	−77
HIV/AIDS and tuberculosis	6743.0 (4781.7–9055.3)	3272.7 (2377.7–4478.2)	−51%
Diarrhea, lower respiratory infections, and other common infectious diseases	49,543.4 (39,838.4–64,637.7)	8564.2 (6756.2–10,664.7)	−83%
Neglected tropical diseases and malaria	5356.8 (2041.4–10,415.4)	327.1 (194.2–527.3)	−94%
Maternal disorders	2078.0 (1564.4–2721.2)	720.7 (306.9–1553.6)	−65%
Neonatal disorders	16,780.3 (14,338–19,089.8)	5363.7 (4629.4–6102.9)	−68%
Nutritional deficiencies	6466.0 (2263.7–12,308.2)	1131.2 (574.9–1966.7)	−83%
Other communicable, maternal, neonatal, and nutritional diseases	3229.6 (1591.0–5438.4)	1365.1 (781.2–2141.7)	−58%
Non-communicable diseases	16,778.9 (13,317.8–19,893.4)	8823.0 (5926.8–12,768.2)	−47%
Neoplasms	2572.0 (2087.7–3109.5)	1798.1 (1049.8–2962.1)	−30%
Cardiovascular diseases	5188.9 (4328.9–6166.2)	2586.0 (1582.8–4029.0)	−50%
Chronic respiratory diseases	985.9 (774.6–1228.7)	378.5 (239.5–574.9)	−62%
Cirrhosis	1000.4 (784.3–1220.3)	438.1 (251.3–716.6)	−56%
Digestive diseases	1341.3 (974.2–2078.4)	504.4 (306.6–805.1)	−62%
Neurological disorders	560.1 (352.9–820.1)	312.2 (197.0–475.8)	−44%
Mental and substance use disorders	105.9 (77.3–146.9)	58.8 (30.4–108.9)	−44%
Diabetes, urogenital, blood, and endocrine diseases	1760.5 (1235.1–2468.3)	892.7 (571.7–1348.2)	−49%
Musculoskeletal disorders	18.8 (13.7–24.8)	15.6 (8.2–28.2)	−17%
Other non-communicable diseases	3245.1 (1843.7–5033.3)	1838.6 (1034.3–2469.9)	−43%
Injuries	13,357.6 (8491.7–18,561.4)	2829.4 (1910.7–4161.7)	−79%
Transport injuries	1593.1 (989.6–2294.4)	709.7 (455.5–1108.5)	−55%
Unintentional injuries	4414.5 (2303.9–6511.1)	1439.1 (981.5–2077.8)	−67%
Self-harm and interpersonal violence	1068.8 (745.8–1840.0)	659.2 (366.7–1186.6)	−38%
Forces of nature, war, and legal intervention	6281.1 (2288.3–10,983.1)	21.4 (6.1–40.8)	99.7

All Level 2 causes showed reductions in crude YLL rates. CMNN diseases declined between 51 and 94% (Table 3). The two leading causes of NCD-related YLL rates decreased as well. Cardiovascular diseases decreased by 50% and neoplasms by 30%. The leading causes due to injuries also decreased. Unintentional injury declined by 67% and transport injury by 55% (Table 3).

### Age-standardized YLL rates and percentage changes

In 2015, the all-cause age-standardized YLL rate was 35,803.9 (95% UI: 26,058.9–49,095.4), of which CMNN diseases caused 17,950.6 (95% UI: 14,377.9–22,768.8) and NCDs caused 14,761.9 (95% UI: 9422.7–22,306.4) YLLs per 100,000 people (Table 4). The age-standardized YLL percentage contribution of CMNN diseases was 60% in 1990 and 51% in 2015, whereas NCDs contributed 27% in 1990 and 41% in 2015 (Fig. 4). The age-standardized YLL rates declined by 69% for CMNN, 76% for injuries, and 44% for NCDs. CMNN diseases were the leading causes of age-standardized YLL rates in 2015, as indicated in Fig. 4.

**Table 4:** Age-standardized YLL rates per 100,000 peoples for both sex and all age groups with level one and two categories, from 1990 to 2015

Causes of YLLs	1990	2015	AS YLL % change, 1990–2015
All cause	97,461.6 (89,128.7–106,696.7)	35,803.9 (26,058.9–49,095.4)	−63%
CMNN diseases	58,288.6 (54,014.3–63,214.7)	17,950.6 (14,377.9–22,768.8)	−69%
HIV/AIDS and tuberculosis	9005.0 (6606.5–11,606.6)	4240.7 (2936.7–5927.9)	−53%
Diarrhea, lower respiratory, and other common infectious diseases	30,626.1 (25,637.1–38,380.0)	7589.0 (5799.7–9935.2)	−75%
Neglected tropical diseases and malaria	3635.1 (1734.1–6477.5)	281.8 (169.3–449.7)	−92%
Maternal disorders	2498.2 (1880.6–3299.4)	785.4 (330.8–1742.1)	−69%
Neonatal disorders	6710.6 (5734.9–7637.5)	3159.7 (2727.0–3595.2)	−52.9
Nutritional deficiencies	4098.7 (2017.1–6922.2)	983.4 (577.6–1540.4)	−76%
Other communicable, maternal, neonatal, and nutritional diseases	1714.9 (954.5–270.4)	910.6 (557.1–1375.8)	−47%
Non-communicable diseases	26,282.6 (22,553.6–29,989.90	14,761.9 (9422.7–22,306.4)	−44%
Neoplasms	4584.7 (3828.6–5443.7)	3106.9 (1778.6–5136.1)	−32%
Cardiovascular diseases	11,073.6 (9393.1–12,898.8)	5930.7 (3686.2–8993.2)	−46%
Chronic respiratory diseases	1727.1 (1437.7–2049.1)	751.5 (467.3–1144.7)	−56%
Cirrhosis	1764.0 (1390.0–2134.9)	802.7 (462.1–1306.9)	−55%
Digestive diseases	2126.6 (1740–2632.9)	887.2 (540.7–1375.3)	−58%
Neurological disorders	777.3 (597.1–986.6)	497.5 (318.9–750.6)	−36%
Mental and substance use disorders	163.6 (118.7–226.8)	86.1 (44.0–163.7)	−47%
Diabetes, urogenital, blood, and endocrine diseases	2417.7 (1975.9–2958.3)	1437.3 (892.2–2226.4)	−41%
Musculoskeletal disorders	28.3 (21.4–35.9)	22.4 (11.0–43.0)	−21%
Other non-communicable diseases	1619.6 (9,89.7–2416.1)	1239.5 (725.4–1632.1)	−23%
Injuries	12,890.4 (8431.7–17,677.2)	3091.5 (1987.7–4790.8)	−76%
Transport injuries	1641.4 (1185.5–2103.9)	782.0 (485.4–1270.8)	−52%
Unintentional injuries	3776.6 (2653.1–4942.7)	1517.5 (1019.0–2248.3)	−60%
Self-harm and interpersonal violence	1319.2 (9,44.9–2150.2)	771.9 (424.0–1403.1)	−42%
Forces of nature, war, and legal intervention	6153.3 (2258.8–10,730.9)	20.1 (6.1–36.9)	99.7%

**Fig. 5:**
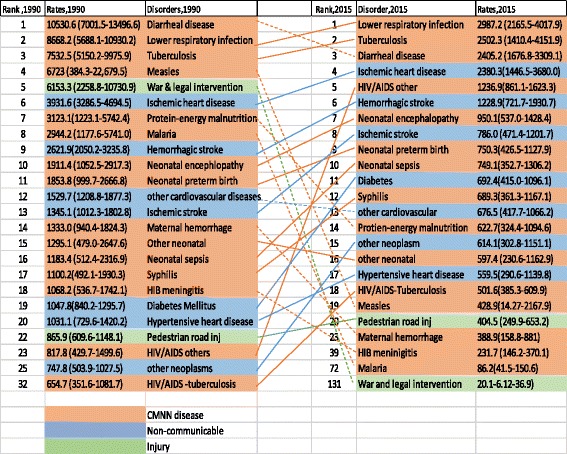
Ranks of causes of age-standardized YLL rates for both sexes and all age groups in Ethiopia, 1990–2015

The top 10 leading causes accounted for 45% of the total age-standardized YLL rate in 2015 (Fig. 5). Seven of the 10 leading causes were CMNN diseases. The top five leading causes were lower respiratory infections, diarrheal diseases, tuberculosis, ischemic heart disease, and HIV/AIDS, causing 2987.2 (95% UI: 2165.5–4017.9), 2502.3 (95% UI: 1410.4–4151.9), 2405.2 (95% UI: 1676.8–3309.1), 2380.3 (95% UI: 1446.5–3680.0), and 1236.9 (95% UI: 861.1–1623.3) per 100,000 people (Fig. 5 and Table 4).

The combined HIV/AIDS and tuberculosis age-standardized YLL rate declined 53%, while diarrhea, lower respiratory infections, and other common infectious diseases declined 75%, collectively. The two leading causes of NCDs, cardiovascular diseases and neoplasms, declined by 46 and 32%, respectively. Leading causes of injuries also declined, unintentional injury by 60% and transport injury by 52% (Table 4).

**Fig. 6:**
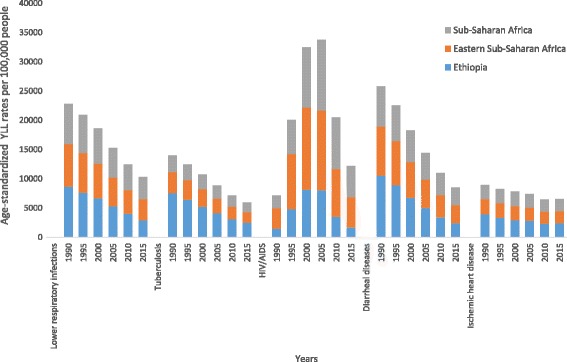
Levels and trends of the top five leading causes of age-standardized YLL rates per 100,000 people for Ethiopia compared with the Eastern sub-Saharan Africa sub-region and the sub-Saharan Africa region

**Fig. 7:**
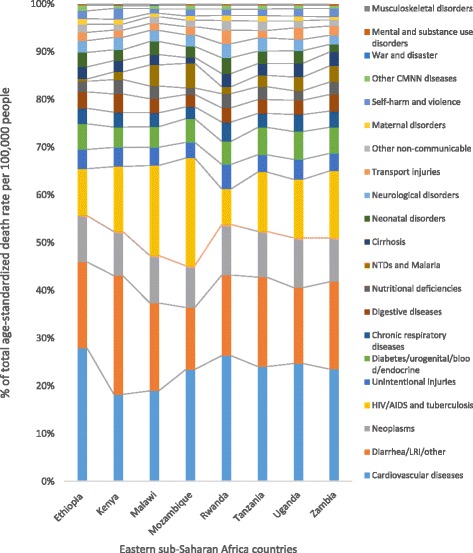
Percentage contribution of diseases to total age-standardized death rate per 100,000 people for both sexes and all age groups in selected Eastern sub-Saharan African countries, 2015

**Fig. 8:**
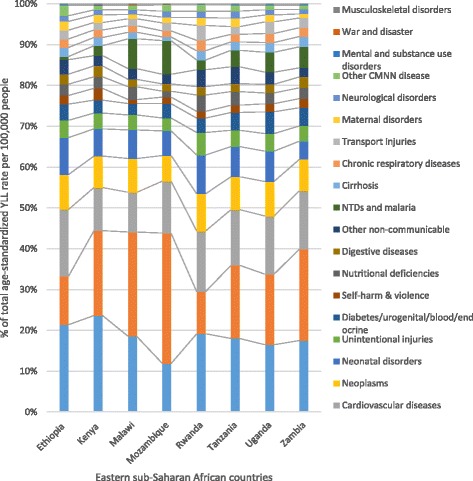
Percentage contribution of diseases to age-standardized YLL rates per 100,000 people for both sexes and all ages in selected Eastern sub-Saharan African countries, 2015

The five leading causes of age-standardized YLL rates for Ethiopia generally showed greater declines when compared to the Eastern sub-Saharan Africa sub-region and the sub-Saharan Africa region; however, ischemic heart disease did not follow this pattern, as indicated in Fig. 6. The contribution of diseases to the total age-standardized death rate and the YLL rate per 100,000 in Eastern sub-Saharan African countries showed variation, as indicated in Figs. 7 and 8.

## Discussion

The age-standardized death rate point estimates for Ethiopia between 1990 and 2015 due to communicable, maternal, neonatal, and nutritional causes have remarkably declined by 65%, from 1257.1 (95% UI: 1128.3–1395.0) per 100,000 in 1990 to 445.0 (95% UI: 326.7–600.7) in 2015. Injury-related causes of age-standardized death rate point estimates have also decreased by 70%, 304.3 (95% UI: 222.0–390.1) per 100,000 in 1990 to 92.2 (95% UI: 59.1–140.6) in 2015. In contrast, NCDs declined by only 37% between 1990 and 2015, 1131.6 (95% UI: 981.7–1278.6) per 100,000 to 710.9 (95% UI: 468.8–1036.2). Regardless of all the above success in reducing CMNN diseases, Ethiopia was not able to achieve sufficient reduction of maternal mortality and neonatal mortality rates to meet the Millennium Development Goal targets [23–25]. This could be due to low coverage of certain cost-effective and proven strategies to reduce maternal and neonatal mortality, such as skilled birth attended delivery, which was below 30% in 2016 [26]. Based on 2015 point estimates, non-communicable diseases were the leading contributor to age-standardized death rates in Ethiopia, causing 710.9 (468.8–1036.2) deaths per 100,000; however, CMNN diseases were leading causes of premature mortality, with a rate of 17,950.6 (14,377.9–22,768.8) per 100,000. CMNN diseases caused more deaths in young people, those between 15 and 49, than other causes in 2015.

In Ethiopia, the top five leading causes of age-standardized premature mortality and death rates in 2015 were lower respiratory infections, tuberculosis, diarrheal disease, ischemic heart disease, and HIV/AIDS. Despite being the leading causes of premature mortality, lower respiratory infections, tuberculosis, and diarrheal disease premature mortality rates declined faster in Ethiopia compared to the overall sub-Saharan Africa region and Eastern sub-Saharan Africa. HIV/AIDS also declined by 70% after the introduction of free antiretroviral therapy (ART) in 2005 [27]. Moreover, age-standardized death rates for neonatal encephalopathy, iron-deficiency anemia, protein-energy malnutrition, and preterm birth complications decreased by 50% or more in Ethiopia between 1990 and 2015.

Comparing this study’s findings with the Ministry of Health’s (MOH) 2015 Health Related Indicators Report [28], there were some variations with the top 10 leading causes of mortality. For example, diarrheal disease was not listed as a leading cause of mortality in the MOH report – the difference could be related to methodology and/or data sources. The MOH used Health Management Information System data as the main data source for health-related indicators, which was not incorporated into GBD 2015 since it does not cover the whole population or capture all disease [16]. Second, disease classification differences could impact the results as health and health-related indicators used larger categories such as cardiovascular diseases and trauma, whereas GBD 2015 used a hierarchical disease categorization system. Third, the 95% UI in GBD 2015 showed overlap in the cause ranks that might affect the comparison, and the noise-reduction process in the estimation may have affected GBD estimates [16].

The findings of this study highlight the epidemiological transition that is happening in Ethiopia: a disease burden transition from predominantly infectious diseases to non-communicable diseases [29]. As a result of this transition, ischemic heart disease, hemorrhagic stroke, ischemic stroke, diabetes, and hypertensive heart disease were found to be among the 10 leading causes of age-standardized death rates in 2015 in Ethiopia. This trend showed that, generally, non-communicable diseases had existed as problems over decades but became more visible following greater reductions in common infectious, maternal, and nutritional diseases. This could largely be explained by population growth and aging: these two factors increase the numbers of deaths from non-communicable diseases in the country as declines in age-standardized death rates are counterbalanced by population growth and aging [21]. The NCD findings suggest heterogeneity in the burden of cardiovascular diseases and diabetes between rural and urban populations. These two populations have diverse sociodemographic, lifestyle, and health risk factors that may vary; however, further subnational exploration is needed [30].

In this phase of the transition, Ethiopia is facing a double disease burden from common infection diseases such as lower respiratory infections, diarrhea, tuberculosis, and HIV/AIDS, as well as from non-communicable diseases including cardiovascular diseases, cancer, and diabetes. These findings support claims that the double burden of NCDs and CMNN diseases exists in Ethiopia [16, 29–33]. In addition, high premature mortality from CMNN diseases would lead to high economic and development challenges in the country [34].

Ethiopia’s success in decreasing the mortality rates of lower respiratory infections, diarrheal diseases, tuberculosis, HIV/AIDS, measles, malaria, protein-energy malnutrition, and other diseases supports the directions and investment of the HSDP on MDGs [2]. However, the plan appears to be unsuccessful in reducing cardiovascular disease, diabetes, and other non-communicable diseases. Non-communicable disease mortality rates have been relatively stable over the years. This might be due to the fact that cardiovascular disease, cancer, diabetes, and/or other non-communicable diseases were not national priorities in Ethiopia as national strategies and action plans targeting non-communicable diseases were not in place until 2010 [13].

The general limitations of the GBD approach also apply to this paper. These limitations have been discussed widely and in detail in the published GBD 2015 articles; however we summarize the relevant limitations focusing on data sources for Ethiopia [7, 11, 19, 21, 35]. Regardless of rigorous and standardized methodology in estimating causes of death, cause-specific mortality data incorporated into GBD 2015 were very scarce for Ethiopia. Causes of death data sources were mainly from verbal autopsy and sibling history. Verbal autopsy data sources in Ethiopia lack national representativeness, whereas sibling history data sources only address maternal health and related estimates. Because of the lack of completeness of these sources, there were wider uncertainties, as indicated with the 95% UI for age-standardized death rates which largely affect policy debates, prioritization of causes, and health decisions.

Moreover, there are limitations with verbal autopsy data sources that affected uncertainty of the estimates. There could be variations in verbal autopsy data-collection methods within and among data sources, including, but not limited to, differences in recall period (between the time of death and interview), the type of questionnaire used, interviewers and physician reviewers, and completeness, which had led to low comparability of data [18, 31, 33, 35–38]. All verbal autopsy data sources represented subnational locations, which greatly affects the uncertainty of national estimates during data processing, data mapping, age-sex splitting, and cause of death redistribution to provide national estimates [17]. The studies including data from demographic surveillance sites were poorly generalizable to the national population; however, the contribution of demographic surveillance sites in GBD 2015 was critical [16].

Verbal autopsy studies usually address child mortality and few types of causes of death [39]. Addressing and reporting all age groups and all possible causes of death including maternal causes of deaths and the current emerging non-communicable diseases among adults in Ethiopia would improve data quality [14]. Moreover, GBD 2015 mainly used verbal autopsy studies from scientific literature, which lack comprehensiveness and detail in reporting causes as they report broad age categories and lack reporting of cause of death distribution by sex and others. This resulted in excluding some data sources or including the data at a higher cause level. This may require close interaction with actual data sources to access comprehensive and detailed data for GBD estimates.

In addition, changes in cause of death due to rapid urbanization as well as demographic and epidemiological transitions with subnational and rural-focused verbal autopsy studies are challenging.

### Implications of the findings for policies and practices

Primarily, the findings are useful to highlight the performance of the previous health sector development plan for Ethiopia and to create benchmarks for the health sector transformation plan [12]. These findings are also helpful for revisiting some of the strategies and budget reallocations in order to incorporate more data on health and the demographic and epidemiological transitions happening throughout the country. However, findings with wider 95% UIs need to be cautiously considered during policy debates and priority-setting. For example, the uncertainty intervals for age-standardized death rates caused by non-communicable disease were 14,761.9 (95% UI: 9422.7–22,306.4) and for CMNN diseases, 17,950.6 (95% UI: 14,377.9–22,768.8); this shows lack of adequate and quality data due to the wide interval. With regard to the leading causes of age-standardized premature mortality rates, HIV/AIDS has relatively narrow uncertainty intervals, whereas lower respiratory infections, tuberculosis, diarrheal diseases, and ischemic heart disease have shown relatively wider uncertainty intervals. The health burden variation between urban and rural populations and between regional states and districts requires further exploration to support public health policy at each level.

### Progress in combating common infectious diseases

There was significant progress in reducing premature mortality and death from lower respiratory infections, diarrheal diseases, tuberculosis, and HIV/AIDS in Ethiopia for the past two and a half decades; however these diseases are still large drivers of premature mortality and death. Vaccine-preventable diseases such as measles, whooping cough (pertussis), and tetanus have largely decreased, showing the effectiveness of the health care system and vaccination campaigns. This was instrumental in reducing child mortality in Ethiopia [2]. However, the findings may vary with subnational regional states and/or urban/rural populations which GBD 2015 did not capture. This provides one possible direction for future collaborations with the nine autonomous regional states and two city administration councils in Ethiopia.

### Priority for non-communicable diseases

The different types of cardiovascular diseases, such as ischemic heart disease, hemorrhagic stroke, ischemic stroke, and hypertensive heart disease, were leading causes of premature mortality and age-standardized death rates in Ethiopia. Cervical cancer, diabetes, chronic respiratory diseases, and asthma were also among the leading causes of death and premature mortality rates. These causes are all recent phenomena among Ethiopians; these findings can assist in designing new public health strategies and reinforce recently developed strategies and interventions on cardiovascular disease, cancer, diabetes, and chronic respiratory diseases [13].

### Implementing strategies, strengthening and integrating health services

Our findings could reinforce the implementation of cost-effective strategies, improve resource allocation, and strengthen partnership and community engagement around cardiovascular disease, diabetes, and cancer prevention and control in Ethiopia [40]. In recent years, the MOH and its partners have developed national strategies and action plans focusing on cardiovascular disease, cancer, diabetes, chronic respiratory diseases, and mental health; however, there are implementation challenges. These challenges include, but are not limited to, human resource capacity, financial resources, and the lack of local and global advocacy. There is also a need to reinforce efforts and initiatives on cervical cancer screening, hypertension screening, diabetes screening, HBV vaccination, and media advocacy regarding leading causes of premature mortality and deaths for Ethiopia [12].

Health services that target cardiovascular diseases, cancers, chronic respiratory diseases, and diabetes need to be integrated within the existing primary health care system. Furthermore, these services need to extend to the health post, health extension program, and secondary and tertiary health facilities [2]. Establishing centers and a proper referral system would be helpful, such as oncology centers, cardiac centers, and mental health centers in the secondary and tertiary hospitals. Additionally, there is a need to involve the private sector and civic society in delivering health services for cardiovascular disease, cancer, diabetes, and their risk factors. Moreover, human resource capacity development both in the pre-service and in-service sectors need to consider addressing the double burden of common infectious diseases such as lower respiratory infections, diarrheal diseases, tuberculosis, and non-communicable diseases like cardiovascular disease, cancer, diabetes, and their risk factors. To succeed, essential medical technologies and treatment for cardiovascular disease, cancer, and diabetes are needed to strengthen the health system.

Ethiopia has already ratified the WHO Framework Convention on Tobacco Control (FCTC) and the Protocol to Eliminate Illicit Trade in Tobacco in 2015 [41]; however, it is important to develop implementation guidelines and regulatory mechanisms that can enforce the convention in order to reduce the population’s risk of developing cardiovascular disease, cancer, diabetes, and chronic respiratory diseases. Reducing tobacco use by establishing tobacco-free environments, increasing public knowledge of the hazards of tobacco, and continuously increasing taxes on purchase and sales of tobacco products are recommended interventions in the convention. There is also a need to develop and implement guidelines on alcohol use in Ethiopia as excessive alcohol use is widely prevalent in Ethiopia [15, 42].

### Multi-sectoral response against non-communicable disease

The impact of the different types of cardiovascular disease, cancers, chronic respiratory disease, asthma, and diabetes, as well as their risk factors, could affect sectors beyond the health sector. Therefore, the response for the prevention and control of these leading non-communicable diseases require a multi-sectoral approach. Multi-sectoral strategies have been widely practiced to respond to the HIV/AIDS epidemic in Ethiopia. This expertise would be useful in combating non-communicable diseases such as cardiovascular disease, cancer, diabetes, and their risk factors in Ethiopia. This strategy should involve a variety of sectors such as health, agriculture, trade and industry, education, urban planning, and transportation. These sectors can be coordinated to prevent and control the specified diseases based upon their shared risk factors including tobacco, physical inactivity, unhealthy diet, and excessive alcohol use, as well as “Khat” consumption [42]. Therefore, a collective multi-sectoral response and integration with the existing health care system will allow for the mobilization of resources, foster strong political commitment, and foster a strong collaboration with NCD stakeholders, ultimately addressing the burden of cardiovascular disease, cancer, diabetes, and chronic respiratory disease in Ethiopia. Initiatives on cardiovascular diseases, cancer, diabetes, and chronic respiratory diseases need to adapt some relevant and successful strategies from CMNN initiatives such as HIV/AIDS, tuberculosis, and diarrheal disease to design cost-effective packages.

### Ensure quality data availability and accessibility

The 95% UI for premature death and death rate estimates varies by causes that positively or negatively affect health policy debates and decisions for Ethiopia. Although attaining good-quality vital statistics could be a long-term goal for Ethiopia, an interim solution must be devised. Investing in developing a national data management center and developing a national data-sharing policy and culture that would pool all data sources available in the country would help to provide more accurate burden of disease estimates. This would ultimately better inform policy debates and decisions at national and subnational levels in Ethiopia. Survey and surveillance data and reports need to be released in a timely manner in order to maximize their use and relevance. Moreover, investing in national verbal autopsy studies, such as post-census and/or post-demographic health surveys, would expand health demographic surveillance sites to new locations and would help to fill cause of death data gaps in Ethiopia. Verbal autopsy studies need to capture the different agro-ecological and population structure of the country. Finally, Ethiopia requires higher investment and commitment to generate quality cause of death data through Vital Events Registration Agency (VERA), which was established in August 2016 [43], strengthening and tailoring surveillance systems and surveys toward the inclusion of non-communicable diseases and their risk factors in Ethiopia.

## Conclusions

Ethiopia has been successful in reducing common infectious, maternal, neonatal, and nutritional deficiency causes of premature mortality including lower respiratory infections, diarrheal diseases, tuberculosis, and HIV/AIDS, for the last two-and-a-half decades. Despite this success, lower respiratory infections, diarrheal diseases, tuberculosis, and HIV/AIDS are still large drivers of premature mortality in Ethiopia. The country’s performance on reducing cardiovascular diseases, diabetes, cancer, and other non-communicable diseases has been minimal, causing some non-communicable diseases to become leading causes of death in 2015. Ethiopia should have a strong commitment to implement existing strategies to strengthen and integrate health services as well as design multi-sectoral responses targeting non-communicable diseases. Strategies at various levels should consider the double burden of communicable, maternal, neonatal, and nutritional deficiency diseases and non-communicable diseases. The country should follow short- and long-term strategies to ensure quality data availability and accessibility to measure the health of the nation. Developing a national data management center that pools all available data, collects targeted primary data, and improves uncertainty of the estimates should be a priority. Long-term investment in a vital events registration system and its implementation in the country is crucial. Measuring the burden of diseases at subnational levels and identifying variations between urban and rural populations will be helpful for health policy decisions at different levels.

## Abbreviations

CMNN: Communicable, maternal, neonatal, and nutritional
CODEm: Cause of death ensemble modeling
EDHS: Ethiopia Demographic and Health Surveys
GBD: Global Burden of Disease
HDSS: Health and Demographic Surveillance System
HIV/AIDS: Human Immunodeficiency Virus/Acquired Immune Deficiency Syndrome
HSDP: Health Sector Development Program
MDGs: Millennium Development Goals
NCDs: Non-communicable diseases
ST-GPR: Spatiotemporal Gaussian process regression
UI: Uncertainty intervals
WHO: World Health Organization
YLL: Years of life lost
